# Electronic Immunoassay Using Enzymatic Metallization on Microparticles

**DOI:** 10.1021/acsomega.3c01939

**Published:** 2023-05-24

**Authors:** Josiah Rudge, Madeline Hoyle, Neda Rafat, Alexandra Spitale, Margaret Honan, Aniruddh Sarkar

**Affiliations:** Wallace H. Coulter Department of Biomedical Engineering, Georgia Institute of Technology, Atlanta, Georgia 30332, United States

## Abstract

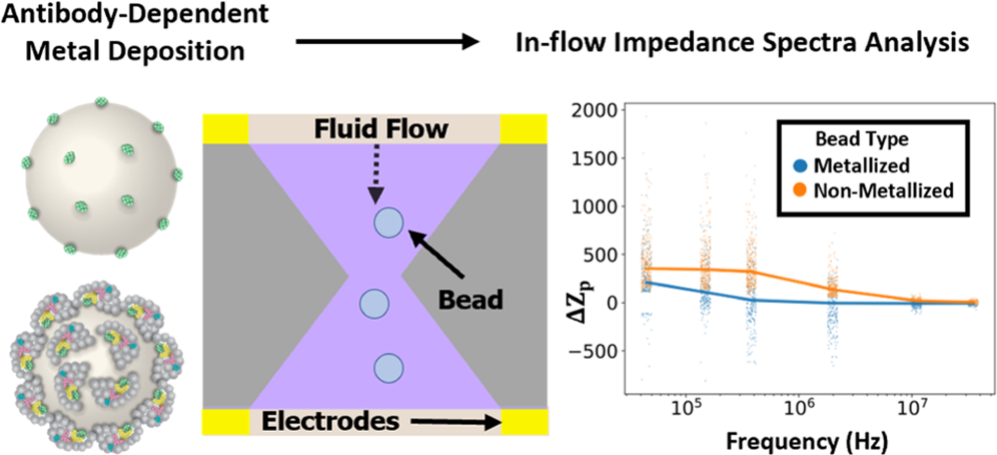

We present here an inexpensive method for generating a sensitive direct electronic readout in bead-based immunoassays without the use of any intermediate optical instrumentation (e.g., lasers, photomultipliers, etc.). Analyte binding to capture antigen-coated beads or microparticles is converted to probe-directed enzymatically amplified silver metallization on microparticle surfaces. Individual microparticles are then rapidly characterized in a high-throughput manner via single-bead multifrequency electrical impedance spectra captured using a simple and inexpensive microfluidic impedance spectrometry system we develop here, where they flow through a three-dimensional (3D)-printed plastic microaperture sandwiched between plated through-hole electrodes on a printed circuit board. Metallized microparticles are found to have unique impedance signatures distinguishing them from unmetallized ones. Coupled with a machine learning algorithm, this enables a simple electronic readout of the silver metallization density on microparticle surfaces and hence the underlying analyte binding. Here, we also demonstrate the use of this scheme to measure the antibody response to the viral nucleocapsid protein in convalescent COVID-19 patient serum.

## 1 Introduction

Bead-based immunoassays provide excellent sensitivity, speed, and extended dynamic range due to their enhanced binding kinetics and ease of automation.^1^ These assays operate by immobilizing a capture antigen on the surface of microscale beads (or microparticles), incubating with the sample of interest, and then binding to the captured analyte with a reporter molecule or system.^2^ Most commonly, this reporter molecule is a secondary antibody labeled with a fluorophore.^3^ This bead-bound complex can be suspended in fluid for easy handling and passed through a laser beam to excite the fluorophore for optical detection.^4^ In-flow serial measurement of thousands of individual beads can thus be performed in minutes. However, the optical detection systems required, such as flow cytometers, are complex, bulky, and expensive as they rely on lasers, photodetectors, and beam-shaping lenses. This hinders their use, especially in the context of widespread infectious diseases such as COVID-19. This raises the question of what other measurable properties could be linked to analyte binding in an immunoassay while maintaining the benefits of bead-based assays and in-flow measurement systems.

Here, we develop an inexpensive, electronic alternative to fluorophores and optics for analyte detection by using a bead-based assay chemistry that mirrors an enzyme-linked immunosorbent assay (ELISA).^5^ However, rather than the enzyme-labeled secondary probe catalyzing a color or fluorescence change in solution, we catalyze a metal deposition^6,7^ onto a bead surface, thus changing the electrical properties of the beads. This enables the use of a simple electrical detection system, based on the “Coulter principle,” to measure the impedance of beads in continuous flow.^8^ Here, an inexpensive in-flow multifrequency impedance characterization system is developed, using a three-dimensional (3D)-printed plastic microaperture to measure impedance characteristics of microparticles which are then linked to an immunoassay via the enzymatic metallization. Using a model binding assay to study the metal deposition and impedance characteristics of metallized beads, we found that they possess a unique impedance spectral signature that enables distinguishing them from nonmetallized beads. Further, corroborated with analytical models and numerical simulations, these impedance signatures were also found to provide quantitative information about the amount of metallization on beads. A machine learning method was then applied to reduce the measured multivariate bead impedance spectra to define a single metric that clearly distinguishes metallized and nonmetallized beads and a limit of detection in terms of this reduced metric was then also measured. Finally, we integrate and apply these methods to a clinical immunoassay by measuring antibodies directed against the nucleocapsid protein of SARS-CoV-2 in convalescent COVID-19 patient serum. COVID+ and healthy samples were clearly distinguished using this assay. Thus, a fully electronic bead-based immunoassay is realized, which we term the **B**ead-based **E**lectronic Bio-**A**ssay **D**etection using **E**nzymatic **M**etallization or BEAD-EM, which provides a sensitive readout of analyte binding without any intermediate optics.

## 2 Experimental Section

### 2.1 Bead-Antigen Coupling

Magnetic and nonmagnetic polystyrene beads functionalized with carboxyl groups were obtained from Spherotech (3.9, 5.6, and 8.2 μm) and Polysciences (4.7, 5.7, 10.3, and 20.1 μm), respectively. Note that while these magnetic and nonmagnetic beads of different sizes are used for characterizing the microfluidic bead impedance measurement system, eventually, only the 8.2 μm magnetic beads are used to perform the immunoassays. To help normalize the surface chemistry stoichiometry between different bead sizes and stock concentrations, the microparticle surface area per solution volume was calculated for each stock solution assuming spherically shaped beads. Stock solution volumes corresponding to 25 cm^2^ of beads by surface area were taken from stock and suspended in 1 mL of a 1:10 dilution of MES buffer. 40 μL of EDC and 40 μL of NHS at 50 mg/mL were added, and this solution was incubated for 20 min on a vortexer. After incubation, two wash cycles were performed with a 1:10 dilution phosphate buffer. Next, 200 μL of 1:10 dilution phosphate buffer is added with the desired antigen at 50 μg/mL and incubated for 2 h on a vortexer. Following this, 2 washes were performed in 1% PBS with 0.1% Tween 20 and 0.1% bovine serum albumin, and the beads were stored in this same solution. We note here that the streptavidin–biotin-based binding of the antigen on the bead was also attempted before choosing the above EDC-NHS chemistry, but this resulted in bead aggregate formation and hence was not pursued further. Additionally, antigen concentration optimization results are shown in Figure S1.

### 2.2 Model Immunoassay

For the initial investigation of the metal deposition, recombinant protein A/G (ThermoFisher Scientific) was immobilized as the antigen. This was incubated for 60 min with 200 nM mouse anti-human IgG Fc-Horse Radish Peroxidase (HRP) (SouthernBiotech) in 100 μL volume. Beads were washed 3 times in deionized water before the silver deposition was performed with EnzMet (Nanoprobes). 50 μL each of EnzMet solutions A, B, and C were incubated with beads for 4, 4, and 8 min, respectively. Note that these times are obtained from assay optimization performed in our prior work with enzymatic metallization.^9^

### 2.3 Clinical Immunoassay

For clinical sample characterization, the SARS-CoV-2 nucleocapsid protein (Sino Biological) was immobilized on the bead surface, followed by incubation with 100 μL of 1:900 dilution of individual COVID-19 patient (*n* = 5) and healthy serum (*n* = 4) (Ray Biotech). This was incubated for 60 min with 200 nM mouse anti-human IgG Fc-HRP in 100 μL volume. Beads were washed 3 times in deionized water before performing silver deposition at the same conditions as above.

### 2.4 Aperture Fabrication

The microscale aperture was fabricated by 3-dimensional two-photon lithography. The geometry of the aperture was digitally defined in CAD software (Fusion 360) and exported for 3D printing by a Nanoscribe Photonic Professional GT2. After laser exposure, the print was developed by a 10 min bath in an SU-8 developer, followed by a 10 min bath in isopropanol and set under a UV lamp overnight before assembly with other components.

### 2.5 Impedance Spectrometry

A lock-in amplifier and transimpedance amplifier (HF2LI, HF2TA, Zurich Instruments) were used for impedance measurements. Impedance spectra were sampled at 57,000 samples per second at 6 frequencies ranging from 45 kHz to 35 MHz. These time-series signals are passed through a high-pass filter before further analysis. For waveform identification, the impedance magnitude at 45 kHz is used in a peak finding algorithm with a threshold of 110 Ω. Timestamps of peaks at this frequency are used to directly index the remaining 5-magnitude and 6-phase time-series measurements. The flow rate of the syringe pump was 10 μL/min. All aggregate measurements reported here are for 60 beads each.

### 2.6 Metallization Metric

To reduce the multidimensional data acquired for many beads in each trial to a single measure corresponding to antibody presence in that trial, a subset of the acquired data was used in a linear discriminant analysis (LDA) model.^10^ To create training data for the model, mIgG-HRP and mIgG-PE were directly conjugated to the beads and incubated with the metallization substrate to create “high-metal” and “low-metal” beads, respectively. The impedance and phase of peaks at 11 MHz were used as features in the LDA model after being scaled to a standard distribution (*z*-score). This model is then used to quantify the limit of detection and clinical assays. Specifically, the model is applied to each of the 60 beads measured in each trial to yield their principal components, and the mean of this is taken to represent that trial and serves as a measure of the degree of metallization on the beads. The Python code used for this analysis is made available at https://github.com/MNBEL/BEAD-EM. For dilutions curves, four parameter logistic (4PL) curves were fit using Prism.

### 2.7 Finite-Element Modeling and Simulations

Finite-element analysis was performed with COMSOL Multiphysics 5.6 with the AC/DC module. To match the baseline of the empirical system, lumped elements were added to the electronic circuit interface. Nonmetallized beads were simulated as nonconducting spheres and metallized beads were simulated with the electric shielding feature on the nonconducting sphere surface.

## 3 Results and Discussion

### 3.1 Enzymatic Metal Deposition on Microparticles

**Figure 1:**
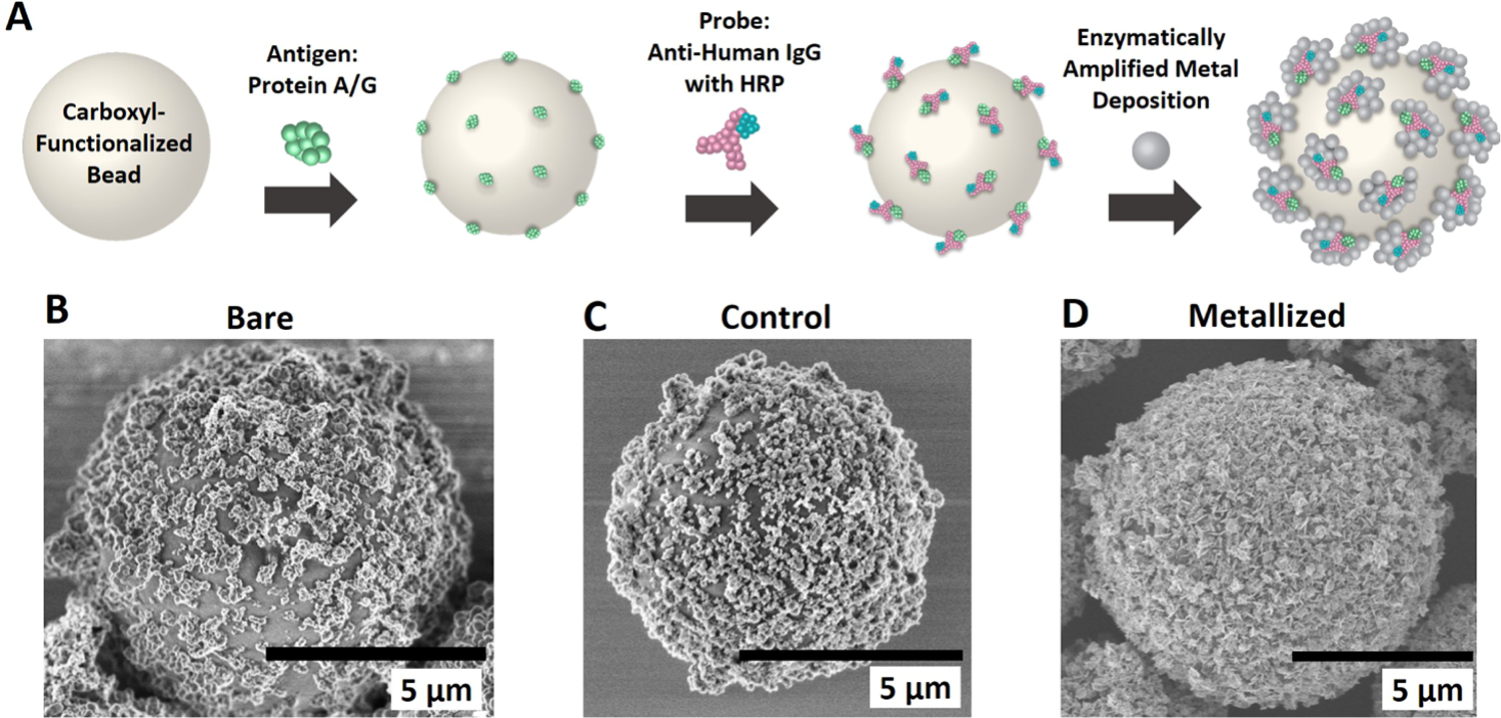
(A) Key steps for immunobinding and enzymatic metallization using a model assay. Protein A/G is attached as an antigen to carboxyl groups by NHS-EDC chemistry. HRP-conjugated antibodies bind to immobilized antigens on the bead surface. The HRP enzyme catalyzes metal deposition. (B) SEM image of a carboxyl-functionalized bead as received from the vendor. (C, D) SEM images of beads after metal deposition, with PBS and mIgG-HRP as probes, respectively.

We first developed a model binding assay to investigate immunobinding and target-probe-driven enzymatic metallization on beads and their effect on their electrical properties (Figure 1A). Carboxylated polystyrene beads were functionalized with recombinant protein A/G, which was used as a model capture antigen for binding enzyme-labeled mouse IgG (mIgG-HRP) as an analyte (see the Experimental Section for details). This was followed by incubation with the metallization substrate solution. The beads were then washed, dried, and imaged using electron microscopy. Figure 1B shows stock beads as received from the vendor, which we observe to have a rough surface which likely contributes to their higher binding capacity, as has been observed earlier.^11^Figure 1C shows the control beads which were incubated with reaction buffer alone (1× PBS), and Figure 1D shows the beads which were incubated with mIgG-HRP before incubation with the metallization substrate solution. A dense nanostructured metal film was observed on the mIgG-HRP bound beads but not on the control beads. It is worth noting here that even the bare beads, as obtained from the vendor stock solution, have a rough surface showing microscale features. The metallized beads, however, have a distinctive nanoscale morphology. At the nanoscale, the metal layer is found to have the morphology of overlapping dense “desert rose”-like structures similar to what has been reported earlier for enzymatic metallization.^12^ Overall, these results establish that enzyme-labeled probe-driven metal deposition on polystyrene beads can be used to create selective and specific immunobinding-driven metallization. The distinctive nanostructured morphology of the metal thin film raises the possibility of a unique electronic signature of metallized beads, which we explore next by building and using a microfluidic system for bead impedance spectrometry.

### 3.2 Microfluidic System for Bead Impedance Spectrometry

**Figure 2:**
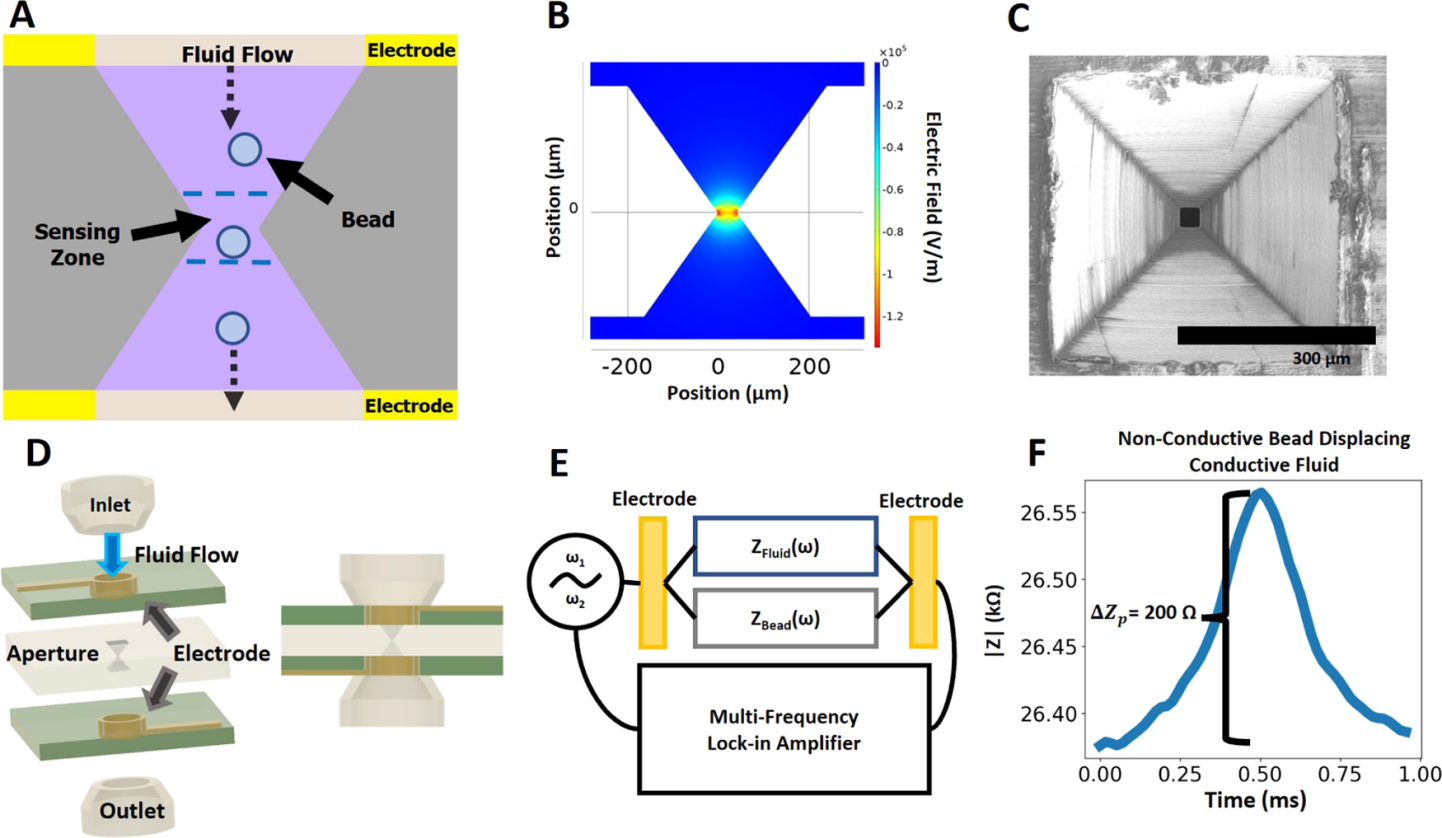
(A) Microfluidic impedance sensing in a tapered aperture. (B) Magnitude of the electric field at the aperture. (C) SEM image of the 3D-printed aperture. (D) Components of the microfluidic system. (E) Equivalent circuit and schematic of the electronic measurement. (F) Example measured impedance “waveform” of a passing polystyrene bead. The peak impedance change, Δ*Z*_p_, is indicated.

Next, we developed and characterized a microfluidic system for high-throughput, sensitive, in-flow impedance characterization of individual beads where the beads in suspension pass through a microscale aperture placed between two plated through-hole electrodes (Figure 2A). The aperture, shaped as an inverted double-pyramid, localizes the electric field to a small region, as evidenced by the COMSOL Multiphysics simulation (Figure 2B). Thus, the impedance measured between the two electrodes is expected to be highly dependent on the electrical properties of a restricted “sensing zone” near the smallest cross-section of the aperture. This allows in-flow measurement of single beads in the sensing zone without cross-talk from other beads in the surrounding fluid. An electron micrograph of the fabricated aperture (top view), which is 30 μm × 30 μm at the narrowest part, is shown in Figure 2C. This image shows one of the two inverted pyramidal faces that form the aperture. The overall sensor assembly is depicted in a schematic in Figure 2D, showing the electrodes, defined as gold-plated through-holes (0.6 mm diameter) on standard printed circuit boards, which are aligned with the aperture and glued together along with fluid connections to a reservoir and connection to a syringe pump that pulls fluid through the aperture. The impedance across the electrodes is measured simultaneously at six frequencies using a lock-in amplifier (Figure 2E). When a bead passes through the aperture, the impedance increases transiently, as shown in an example measured impedance waveform shown in Figure 2F, which appears as a symmetric waveform rising as the bead enters the sensing zone, reaching a maximum or peak value and then falling as it exits.

To characterize the operation of the microfluidic impedance measurement system, the frequency response of the system was measured for various levels of salt concentrations in the buffer fluid flowing through the aperture without any beads (Figure S2). We observe that the system shows a flat (i.e., resistive) magnitude response up until a salt concentration-dependent cutoff frequency, after which it declines, indicating that capacitive effects dominate in that frequency range. Additionally, inductive effects appear at the high-frequency end. Also, as more salt is added, the impedance is found to decrease, as expected, due to the increasing conductivity of the fluid. To better understand these impedance characteristics of the system, a COMSOL impedance model of the system, including the finite-element analysis (FEA) model of the aperture itself, was also built for comparison with fluid conductivity set to that of 1% saline (Figure S3). This model allows the extraction of the lumped impedance parameters representing both the capacitive and inductive parasitic effects, and the fitted model output matches the corresponding measured impedance spectrum, as shown in Figure S2. This coupled FEA and circuit COMSOL model was used hence as the baseline for modeling bead impedances as well. Next, we used this microfluidic system for bead impedance spectrometry developed here to measure the impedance spectra of nonmetallized and metallized beads.

### 3.3 Impedance Spectra of Nonmetallized Beads

**Figure 3:**
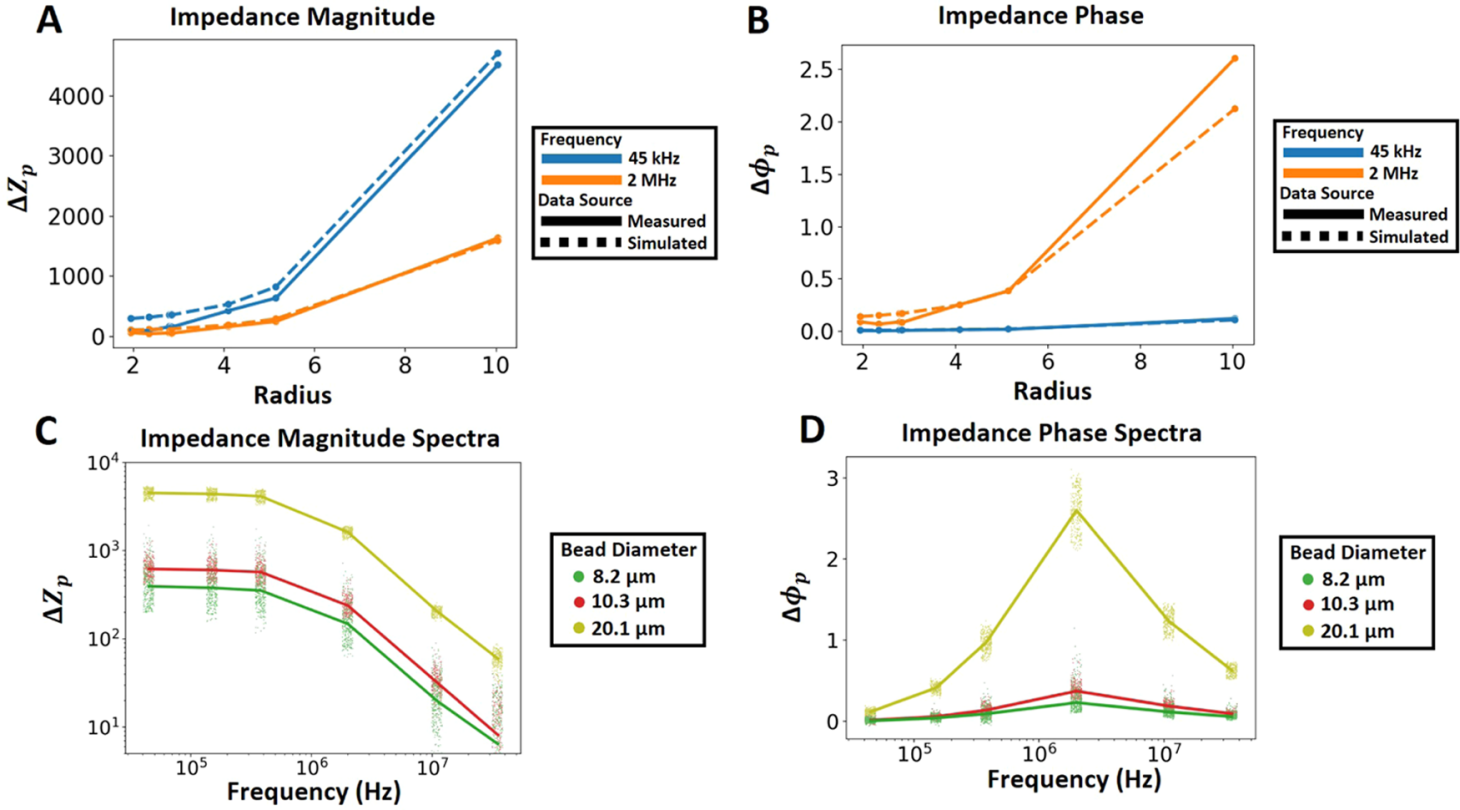
(A, B) Peak impedance (Δ*Z*_p_) and phase change (Δϕ_p_) at two selected frequencies for different bead sizes and corresponding simulation results. (C, D) Impedance and phase change spectrum for different bead sizes. All aggregate measurements reported here are for 60 beads each.

Impedance magnitude and phase waveforms were recorded for seven different sizes of nonmetallized polystyrene beads ranging in diameter from 3.9 to 20.1 μm flowing through the aperture, and the peak change in magnitude (Δ*Z*_p_) and phase (Δϕ_p_) values were obtained for each bead (see the Experimental Section for details). Figure 3A,B (solid lines) shows the variation of Δ*Z*_p_ and Δϕ_p_ with the bead radius at two selected frequencies (45 kHz & 2 MHz). As expected, Δ*Z*_p_ rises with bead size as larger nonconductive beads displace more of the conductive fluid filling the sensing zone. Δϕ_p_ remains low, showing that the impedance change due to beads is mostly resistive at these two frequencies, with larger bead sizes showing slightly higher capacitive phase change. These results were compared with COMSOL simulation results for nonconducting spheres of different sizes passing through the aperture, as shown in Figure 3A,B (dotted lines), which were found to match the measured results.

Figure 3C,D shows the measured full impedance and phase spectra vs. measurement frequency of selected nonmetallized bead sizes. It is observed that Δ*Z*_p_ is flat at lower frequencies but then decreases at higher frequencies. Δϕ_p_ increases and peaks at ∼2 MHz and then declines. Based on these observed spectra, we also built a simplified lumped element circuit model for the nonmetallized beads, which was found to agree well with the measurements (Figure S4). We model the system, without the beads, as a resistance due to the conducting fluid in parallel with capacitance due to the separation of the electrodes. When a bead is added to this, the additional resistance increases the impedance magnitude. A capacitive effect of the bead is also implied by the impedance phase change peaking at 2 MHz and is assumed to be due to the nonconducting bead acting as an additional dielectric between the relatively conductive fluid above and below it. This bead capacitance is modeled in our simple circuit as parallel to the baseline capacitance.

Overall, these nonmetallized bead impedance spectra results and their match with COMSOL simulation results and simplified circuit models establish the ability of the microfluidic system to characterize individual beads in a sensitive and high-throughput manner and distinguish their physical properties such as size. Notably, this system enables this single-bead impedance spectrometry using a simplified 3D-printed microaperture-based sensor assembly without the need for any microelectrodes, microchannels, or associated microfabrication steps.

### 3.4 Impedance Spectra of Metallized Beads

**Figure 4:**
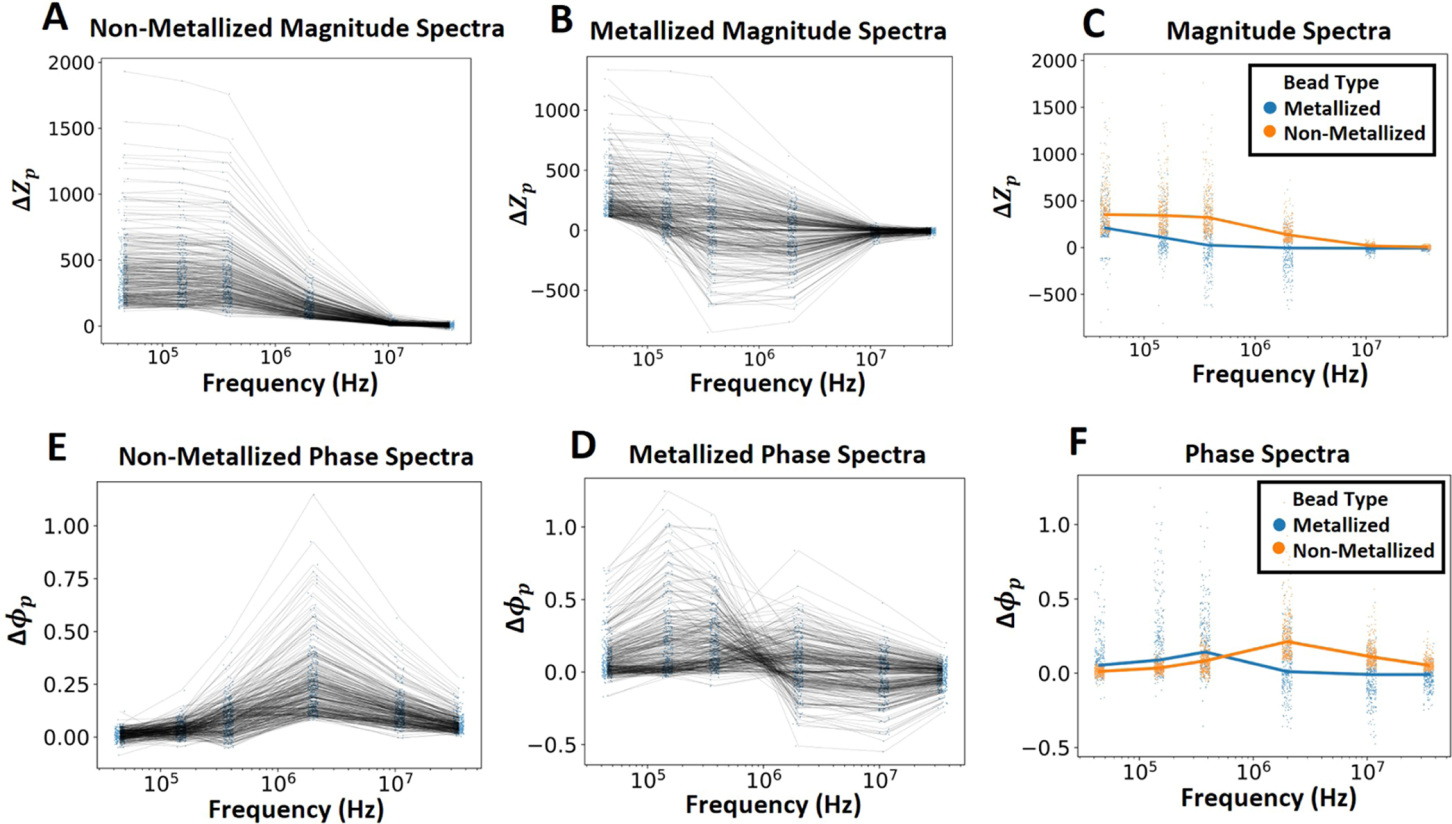
(A, B) Peak impedance change magnitude (Δ*Z*_p_) spectra of nonmetallized and metallized beads. (C) Mean Δ*Z*_p_ spectrum of nonmetallized and metallized beads. (D, E) Peak impedance phase change (Δϕ_p_) spectra of nonmetallized and metallized beads. (F) Mean Δϕ_p_ spectrum of nonmetallized and metallized beads.

Metallized beads (8.2 μm) obtained after enzymatic metallization, as described above, were characterized next. We observed that metallized beads have significantly different impedance and phase spectra compared to nonmetallized beads. Δ*Z*_p_ for metallized beads is, overall, significantly lower than that for nonmetallized beads and even becomes negative at some frequencies (Figure 4A–C). This implies that the metallized beads are more conductive than the surrounding fluid they replace.

However, strikingly, this negative dip is found to be a frequency-specific effect and does not occur across the spectrum. This results in a unique shape of the Δ*Z*_p_ spectra for metallized beads, which is distinguishable from that of the nonmetallized beads. Δϕ_p_ of metallized beads has a positive peak at a lower frequency than the 2 MHz peak of nonmetallized beads and takes on more negative values at 2 MHz (Figure 4D–F). This shows that metallized and nonmetallized beads can be distinguished based on their unique impedance spectra. In the rest of this subsection, we further model, analyze, and explore the unique impedance spectrum of the metallized beads before progressing to using them further in immunoassays.

To better understand the physical basis of these unique impedance characteristics of the metallized beads, in COMSOL, the earlier nonmetallized bead model was altered by adding, around the nonconducting sphere, a thin conductive layer whose conductivity was also varied. We found that in these simulations, at high metal-layer conductivities, negative Δ*Z*_p_ values are indeed predicted. However, the predicted Δ*Z*_p_ values are negative at all frequencies, which is clearly different from the frequency-specific negative impedance effect that is found in measurements. As an example, at high conductivities, Δ*Z*_p_ is simulated to be more negative at 45 kHz than at 2 MHz, but in measurements, metallized beads show the opposite, with negative Δ*Z*_p_ appearing at 2 MHz and not 45 KHz (Figure S5). Therefore, we conclude that the metallized bead surface cannot be modeled simply as a conformal conductive material layer covering the bead surface as such a model does not show the frequency-specific negative impedance signature that is observed for the metallized beads.

**Figure 5:**
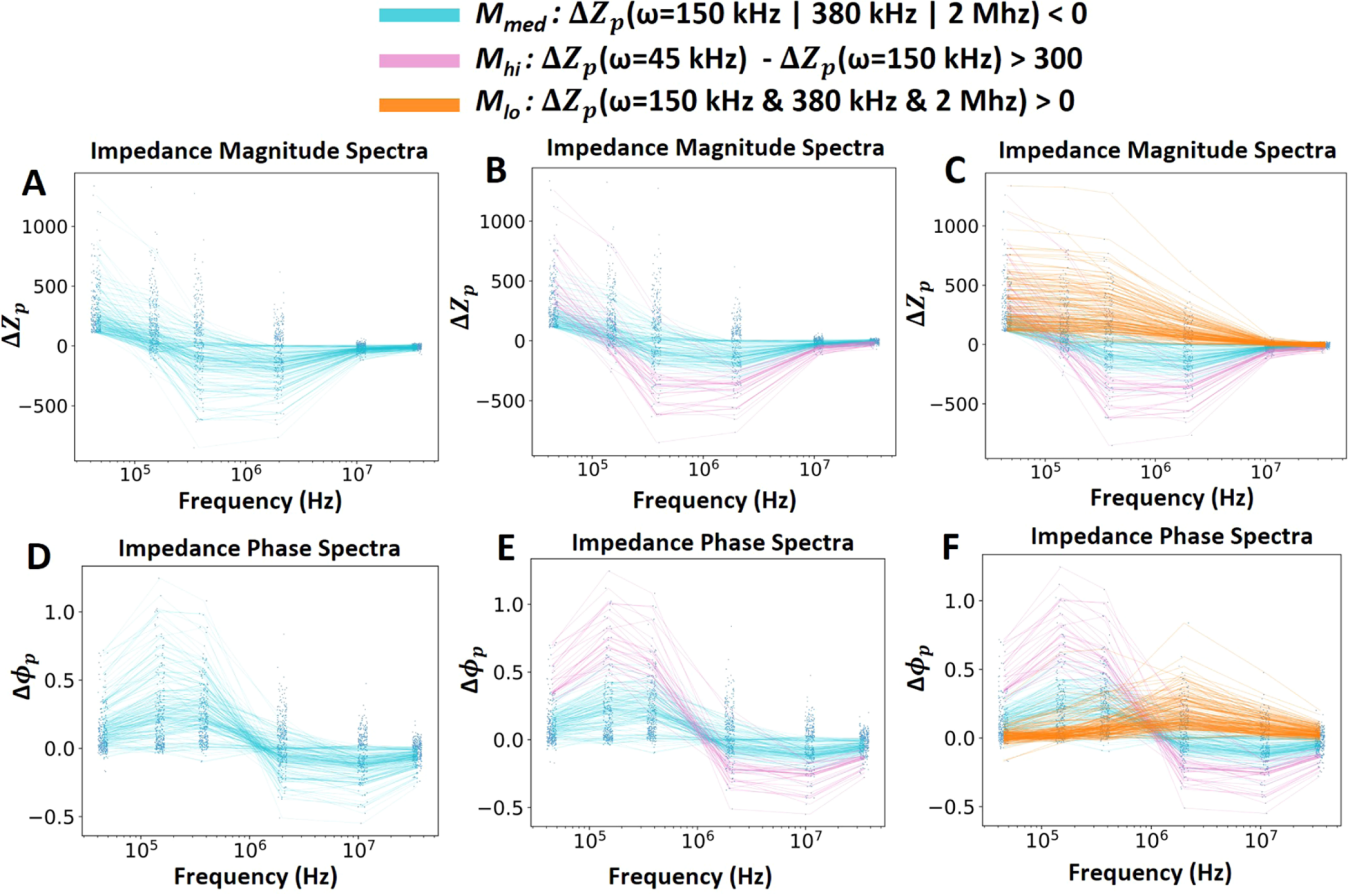
(A) Peak impedance change magnitude (Δ*Z*_p_) spectra of beads with impedance magnitudes less than 0 at 150 khz, 380 kHz, or 2 MHz. (B) Subpopulation from panel A of impedance magnitude spectra with a large decrease from 45 to 150 kHz. (C) Subpopulations in panels A and B overlaid with the remaining population. (D, E, F) Peak phase change (Δϕ_p_) spectra corresponding to populations in panels A, B, and C, respectively.

A lumped element circuit model to match the observed experimental results for metallized beads was developed next. For this, we considered the metallized bead surface as a resistor in series with a capacitor, in parallel to the nonconductive bead circuit model (Figure S6A). This model is found to match the general shape of the impedance and phase spectrum of the metallized beads (Figure S6B,C), including the frequency-specific negative impedance signature. We, thus, intuit that the many individual metal spheroids of a “desert rose”-like shape (Figure 1C) create an intricate mesh of metal and fluid and that an equivalent capacitance may be expected from the metal–electrolyte interfaces and the fluid between conducting metal spheroids instead of a simple conducting layer as modeled in COMSOL earlier. Thus, the distinctive nanostructure of the enzymatic metallization layer may contribute to the unique impedance signature of the metallized beads. Additionally, SEM images of metallized beads showed that there can be significant variation in the degree of metallization of the bead surface (Figure S7). Hence, we explored if the bead impedance spectra contained subpopulations that show different impedance signatures and could be related to variations in bead metallization. Using the impedance spectra of metallized beads from Figure 4, we identify some possible criteria to segregate bead subpopulations, as shown in Figure 5. First, we consider those beads that, at any of the three middle frequencies investigated, have a negative Δ*Z*_p_ (blue curves, Figure 5A–D). This criterion was picked because negative Δ*Z*_p_ is not observed in nonmetallized beads. Furthermore, within this set of beads, we identify a distinct subset that shows a sharp drop-off from relatively large positive Δ*Z*_p_ at the lowest frequency to produce a relatively large negative Δ*Z*_p_ at intermediate frequencies (pink curves, Figure 5B–E). Notably, the Δϕ_p_ for this subset is correspondingly extreme, producing maxima at lower frequencies, followed by a sharper drop-off compared to other beads within the negative Δ*Z*_p_ set. These frequency-specific sharp drop-offs and flip in sign of Δ*Z*_p_ indicate that these metallized beads did not simply undergo a change in overall bead conductivity but demonstrate the unique effect of the nanostructured enzymatic metallization film on the bead surface. We hypothesize that this subset of beads is the one that shows the densest metallization under SEM and term it the *M*_hi_ subset. The rest of the beads in the negative Δ*Z*_p_ set are termed *M*_med_ and are likely the ones with relatively less dense metallization. Figure 5C–F shows *M*_hi_ and *M*_med_ subsets overlaid with the remaining metallized beads. We note that the spectra of these remaining beads from the metallized trial actually match those of nonmetallized beads in Figure 4A–E, and thus we term them the *M*_lo_ beads, which likely undergo very low metallization, which does not result in a conformal metal layer. Overall, the identification of these bead subsets with distinct impedance signatures within the metallized bead impedance spectra indicates the ability of this high-throughput, single-bead impedance spectrometry technique in identifying heterogeneity in the metallization and classifying beads based on it and potentially quantifying such effects as well. As suggested by a reviewer of this work, in future work, elemental analysis using SEM can also be applied to quantify the amount of silver on the beads, and this can be correlated with the impedance readouts.

### 3.5 Metallization Metric and the Limit of Detection

Having developed the bead-based immunobinding, enzymatic metallization, and microfluidic bead impedance spectrometry techniques to generate unique impedance signatures of individual metallized beads, we integrate these into a fully electronic bead-based detection scheme, which we termed **B**ead-based **E**lectronic Bio-**A**ssay **D**etection using **E**nzymatic **M**etallization or BEAD-EM.

**Figure 6:**
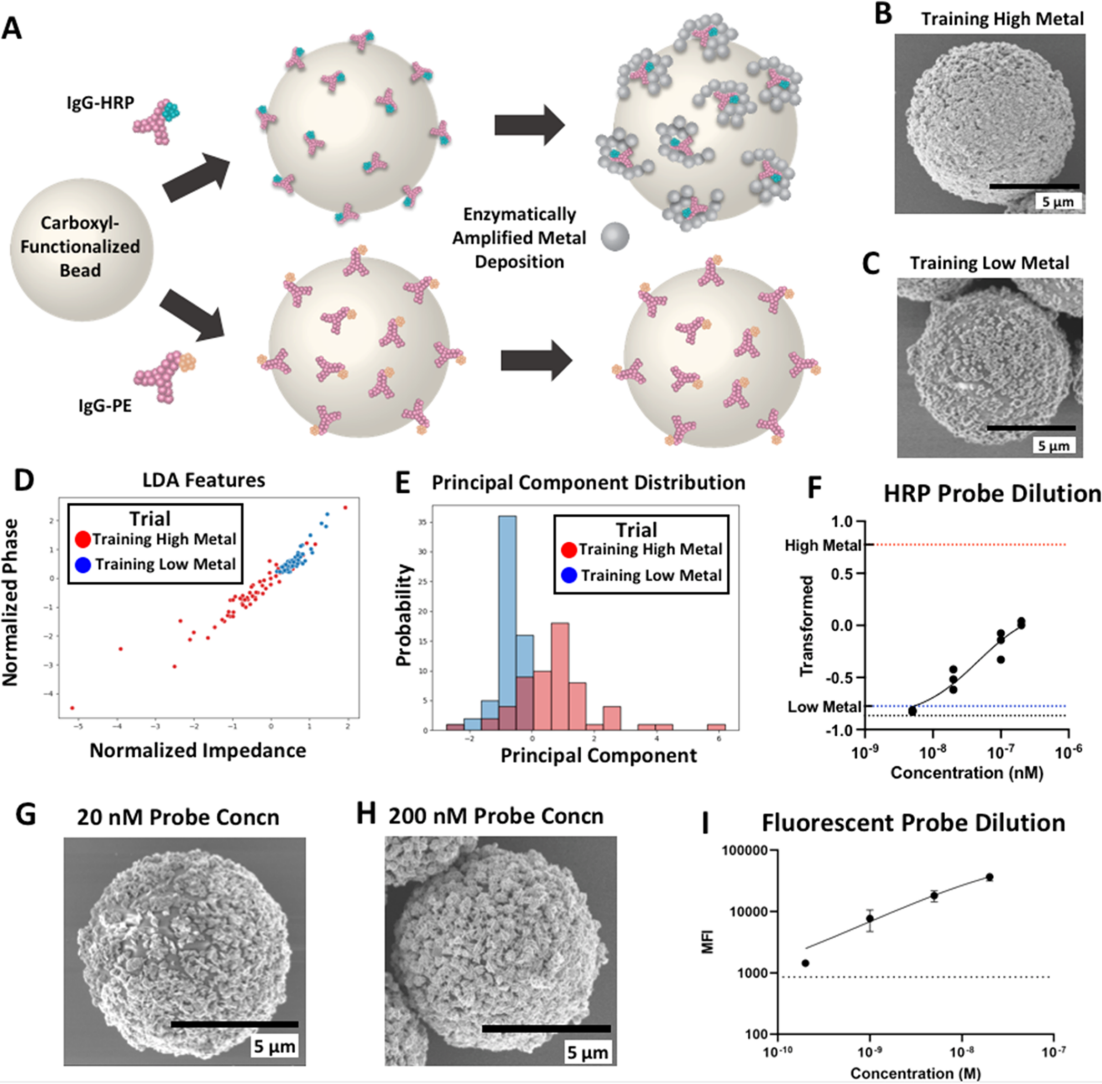
(A) Steps for creating training bead sets. (B, C) SEM images of high-metal and low-metal training beads, respectively. (D) Features used in the LDA classifier. (E) Projection of the principal component. (F) Serial dilution curve (0–200 nM) for obtaining the limit of detection of BEAD-EM for the HRP-labeled probe. The metallization metric for low- (blue dotted line) and high-metal (red dotted line) training sets and the 0 nM probe concentration (black dotted line) is shown. The fitted 4PL curve is also shown. (G, H) SEM images of bead trials at varying probe concentrations. (I) Limit of detection of fluorescent probes measured by traditional flow cytometry. 0 nM trial is shown by the black dotted line. The fitted 4PL curve is also shown.

For the BEAD-EM technique to produce simple-to-interpret results, we decided to build a machine learning model to condense the multivariate impedance spectrum data acquired from each trial into a single “metallization metric.” To generate training data for this model, we conjugated mIgG-PE and mIgG-HRP directly to 8.2 μm magnetic beads and incubated them with a metallization substrate (Figure 6A). This produced beads with low and high amounts of metallization without relying on immunobinding (Figure 6B,C). We chose to use mIgG-PE conjugated beads rather than bare beads as the low-metal training set to confirm that any background metallization or protein immobilization would be included in the model. The 8.2 μm magnetic beads were chosen for this and remaining assays based on a trade-off between a higher signal-to-noise ratio offered by larger bead sizes (Figure 3) versus the increased risk of clogging of the aperture observed with larger beads as well as the ease of handling of magnetic beads (data not shown).

With these low and high metallization samples, a subset of the impedance spectra data (impedance magnitude and phase at 11 MHz) was taken and analyzed with linear discriminant analysis (see the Experimental Section for details), and the principal component of each bead was plotted (Figure 6D,E). The mean of this principal component serves as a single numerical output of the BEAD-EM technique. The impedance measures at 11 MHz were chosen since they produce a particularly stark contrast between high-metal beads, which tend toward negative normalized phase and impedance values, and low-metal beads, which tend toward the opposite direction (Figure 6D).

The limit of detection of the model assay was next determined using this derived metallization metric. The model assay of protein A/G-coated beads probed with mIgG-HRP was performed with 5 different mIgG-HRP concentrations from 0 to 200 nM (Figure 6F). The 20 nM probe concentration was the lowest distinguishable measurement, with a 4-parameter logistic curve fitting predicting a limit of detection (LOD) of 7 nM. SEM images of bead metallization corresponding to 20 and 200 nM are shown in Figure 6G–H, respectively. Measuring the mean fluorescent intensity of fluorescence probes using a flow cytometer, when we used IgG-PE as the probe in our model assay, we found this had a 70-fold lower LOD of 100 pM (Figure 6I). Questions that arise from this result are: Why is the BEAD-EM LOD higher than when using a flow cytometer for detection even in a nearly identical binding assay, and whether and how it can be improved? Additionally, can the current BEAD-EM LOD (∼7 nM) enable its use in specific clinical applications?

Considering the LOD comparison and improvement questions, we expect that the binding constants of mIgG-HRP and mIgG-PE we used are comparable, indicating that this difference in sensitivity is likely due to limitations of either the metal deposition or our measurement technique. With regards to metal deposition, our earlier work^13^ achieved a ∼1–5 pM LOD using a similar metallization reaction on glass slides when using a simplified optical measurement of metallized spot darkness using a cellphone camera in point-of-care immunoassays. However, it is possible that here the changes in the impedance signature of a bead may only occur after significantly higher amounts of metal deposition grow and connect to create a conducting layer. It may be possible to improve this metallization by immobilizing additional catalysts, such as gold nanoparticles, on the bead surface. We have earlier^9^ shown that this technique achieves a ∼4–100 pM LOD for electronic detection of immunoassays on microelectrode arrays.^9^ With regards to the measurement method, sensitivity is highly dependent on the electric field focusing in the aperture and could be enhanced by minimizing its relevant dimensions to more closely match the bead size. Additionally, electrical noise in the system could be reduced if a slower throughput was used, as it would allow the use of a lower cutoff frequency in the low-pass filter inherent in lock-in-amplification. Further, better flow-focusing^14^ of the beads can also reduce variation of impedance introduced by the off-center positioning of some beads in the aperture. It is also worth considering substantial changes to the measurement methodology, such as electrochemical measurement techniques, which have shown high sensitivities to silver on microbeads.^15^ While these remain avenues of future work for the improvement of the assay, we next consider the question of the clinical applicability of the current assay.

### 3.6 Clinical Immunoassay of COVID-19 Biomarkers

We applied the BEAD-EM technique to measure SARS-CoV-2 antigen-specific antibodies from convalescent COVID-19 patient serum. A schematic of the immunoassay is shown in Figure 6A. Beads were functionalized with the SARS-CoV-2 nucleocapsid (N) protein and incubated with individual COVID+ serum (*n* = 5) or prepandemic healthy serum (*n* = 4) as a negative control. It is worth re-emphasizing for clarity here that the beads bound with the N protein are expected to bind anti-N hIgG from serum. These beads were then probed with mIgG-HRP directed against human IgG (hIgG) to measure anti-N hIgG levels in the sera. Finally, they were incubated with the metallization substrate solution. The SEM images in Figure 6B,C show the results of this process. Beads incubated with COVID+ serum show high levels of metallization. We note here again, for clarity, that even the bare beads, as obtained from the vendor stock solution, have a rough surface showing microscale features that is seen on the beads incubated with healthy serum. The metallized beads, incubated with the COVID+ serum, however, have a distinctive nanoscale morphology.

**Figure 7:**
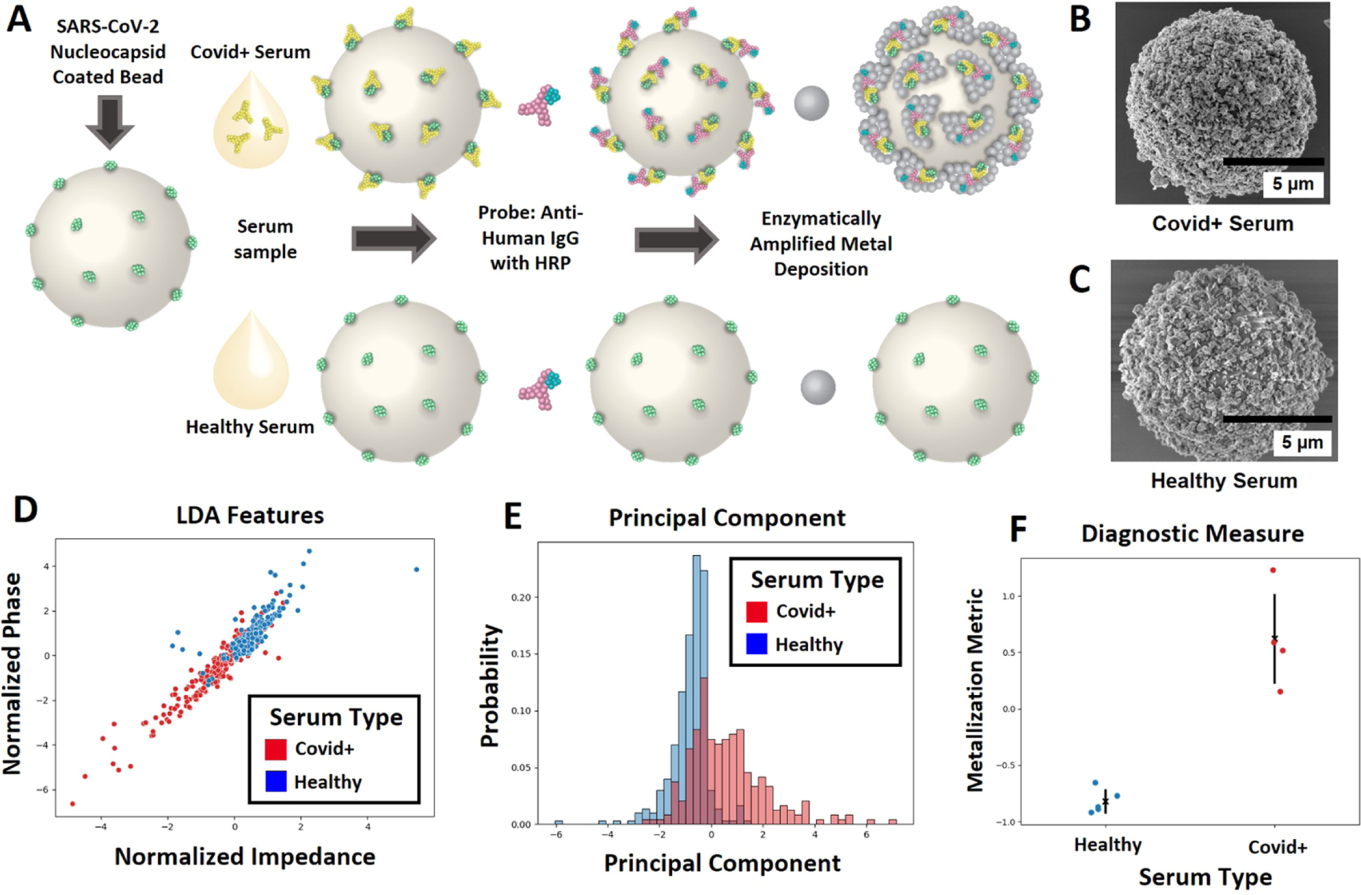
(A) Key steps for the immunoassay for identifying antinucleocapsid IgG in serum from COVID+ patients and healthy prepandemic serum. (B, C) Resulting bead metallization from COVID+ and patient serum, respectively. (D) Features used in the LDA classifier. (E) Projection of the principal component of COVID+ and healthy samples. (F) Final diagnostic measure for COVID+ or healthy serum classification.

After measuring the impedance signatures of the above beads, the same machine learning analysis as above was used to classify the clinical samples while using the same low and high metallization samples training data. The features for the LDA and the resulting distribution of principal components across all clinical samples are plotted in Figure 7D,E, respectively. The mean of the principal components as the final metallization metric of each trial is plotted in Figure 7F, showing clear discrimination between all COVID+ and healthy serum samples. Thus, the BEAD-EM technique enables direct electronic measurement of anti-N IgG as a biomarker of COVID-19 from serum. This establishes that the current BEAD-EM assay has sufficient sensitivity to measure viral antigen-specific antibodies, which are biomarkers of prior infection, can be used to monitor the vaccination status, and can also be used as prognostic markers for monitoring and predicting disease severity.^16^ It is also worth emphasizing for clarity here that these results with clear differences between COVID+ and healthy serum samples inherently establish the specificity of the assay. Additionally, the low metallization metric of healthy serum shows that nonspecific binding from serum does not limit the sensitivity or specificity of this assay.

While the exact concentration of anti-N IgG in patient serum is not known as it can vary based on the stage of infection or host response, it is estimated based on a calibration performed in our earlier work^9^ that a ∼44 pM–1 nM range of antigen-specific IgG concentration is expected in the 1:900 dilution of serum used for the clinical BEAD-EM assay above. Strikingly, this is 7–160-fold below the BEAD-EM LOD measured in the model assay earlier. It is also worth noting here that the 1:900 dilution of serum used for this assay, in fact, leaves significant headroom in the assay dynamic range for even lower concentrations of antibody-based biomarkers as more concentrated serum up to even neat, i.e., undiluted serum is often used in clinical immunoassays. Overall, this result indicates that the sensitivity of the BEAD-EM technique is higher when used with serum than in the model assay. We hypothesize that this may be due to the polyclonal nature of the human antibody response, which may result in more than one epitope on the N antigen being targeted, unlike in the model assay.

## 4 Conclusions

The BEAD-EM assay was developed as a bead-based immunoassay that links analyte binding to enzymatic metallization on bead surfaces to produce “impedance-labeled” beads in contrast to widely used fluorescently labeled beads. A nanostructured “desert-rose” morphology was found for the enzymatic metallization layer on the beads, which forms the basis of the unique electronic signature of metallized beads. In-flow bead impedance sensing was performed using a 3D-printed microscale aperture to capture the impedance spectrum for individual beads in a sensitive and high-throughput yet inexpensive manner. Metallized beads were found to have a distinct impedance spectral signature with frequency-specific negative dips in impedance change which were not found for nonmetallized beads. Thus, metallized and nonmetallized beads could be clearly distinguished using this electronic signature. Additionally, distinct subsets of metallized beads with high, medium, and low metallization-based impedance signatures are found.

A finite-element model for the bead impedance measurement scheme accurately predicted nonmetallized bead impedances but not metallized bead impedances when modeled as nonconductive beads with a purely conductive layer. Instead, a lumped element circuit model, which models the metallization layer as a combined resistive and capacitive element, matches the measured impedance spectra better for the metallized beads. This, we hypothesize, can be linked to the nanoscale structure of the metallization layer.

Finally, the BEAD-EM assay was formalized by using features in the impedance spectra of beads to produce a single quantitative metallization metric. This metric was used to quantify the limit of detection of the BEAD-EM technique in our model assay and compare it to a standard fluorescence-based readout. Furthermore, we applied the BEAD-EM technique in a clinical context to detect SARS-CoV-2 viral antigen-specific antibodies from convalescent COVID-19 patient serum, showing clear differences between COVID+ vs healthy sera. Thus, a clinical immunoassay for electronic biomarker detection without the use of any expensive and bulky intermediate optics was developed and demonstrated.

Overall, while the BEAD-EM assay shows enough sensitivity already to detect antibody-based biomarkers, these results remain a preliminary demonstration of the eventual power and utility of this electronic detection technique. Further work is needed to optimize the sensitivity achieved here, with multiple routes of improvement possible, such as reduction in the heterogeneity of bead metallization, the addition of probe-independent metal deposition catalysts, and fine-tuning of the detection system fluidics and electronics. Additionally, it has not escaped our attention that the unique bead impedance spectra based on both bead size and metallization density could enable the “impedance barcoding” of beads and fully electronic multiplexed bead-based immunoassays in the future.^17,18^ Such a multiplexed BEAD-EM scheme, which remains beyond the scope of this work but could be pursued in future work, would allow the measurement of multiplexed biomarkers, including those that allow prediction of the outcome in severe COVID-19.

## Supporting Information Available

Assessment of antigen conjugation to beads using a BCA assay (Figure S1); impedance spectra of the aperture for varying salt concentrations (Figure S2); schematic of the FEA model connected to ideal circuit components (Figure S3); Bode plot of a simple circuit model of impedance change from a nonconducting bead passing through the aperture (Figure S4); comparison of impedance change between an empirically metallized bead and a simulated conducting bead passing through the aperture (Figure S5); Bode plot of a simple circuit model of impedance change from a metallized bead passing through the aperture (Figure S6); SEM image of several beads showing heterogeneity in metal coverage (Figure S7) (PDF)
