# Supplementary material for: Electronic Immunoassay Using Enzymatic Metallization on Microparticles
Source: ACS Omega. 2023 May 24;8(25):22934–44. doi: 10.1021/acsomega.3c01939 (PMC10308597; doi:10.1021/acsomega.3c01939)
Supplement: Supplementary file 1 — ao3c01939_si_001.pdf [file ao3c01939_si_001.pdf]

Supplementary Material

Electronic Immunoassay Using Enzymatic Metallization on Microparticles

Josiah Rudge, Madeline Hoyle, Neda Rafat, Alexandra Spitale, Margaret Honan, Aniruddh Sarkar*

Wallace H. Coulter Department of Biomedical Engineering, Georgia Institute of Technology, Atlanta GA 30332

*Corresponding author: Aniruddh Sarkar, Email: aniruddh.sarkar@bme.gatech.edu

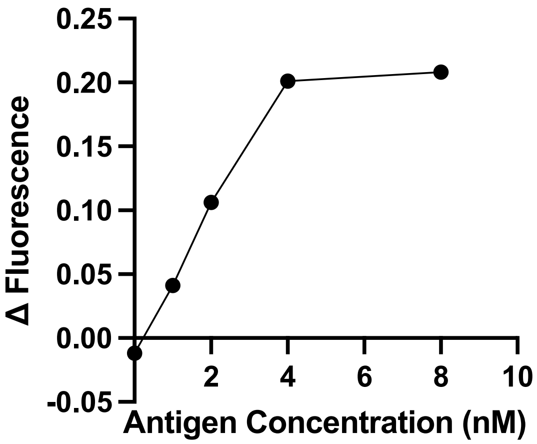

Figure S1: Amount of antigen bound to beads assessed using the difference in pre-conjugation and post-conjugation antigen amounts measured using a fluorescence-based bicinchoninic acid assay (BCA, Thermo Fisher Inc) performed as per manufacturer's recommended protocol.

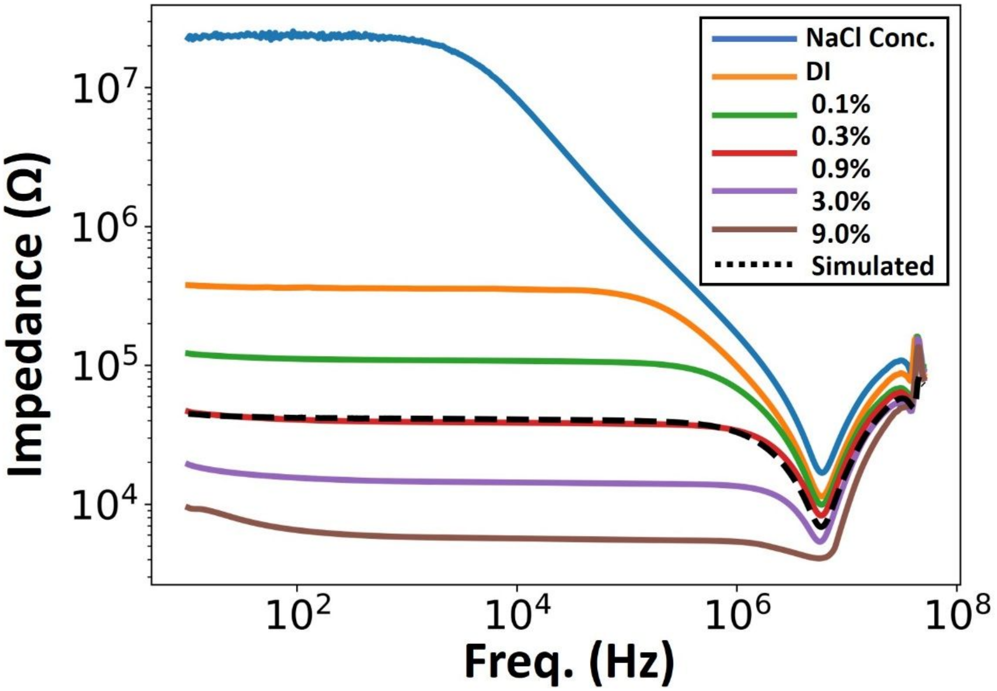

Figure S2: Impedance spectrum for different electrolyte concentrations, and a model matched at our operating NaCl concentration.

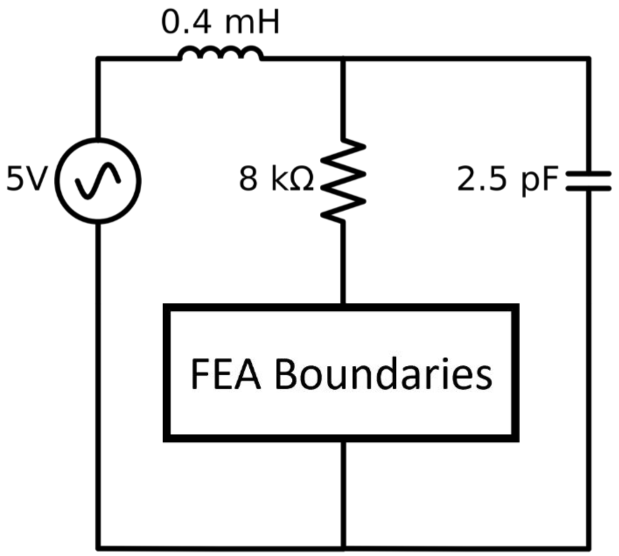

Figure S3: Circuit schematic of simulation setup with ideal components and aperture geometry.

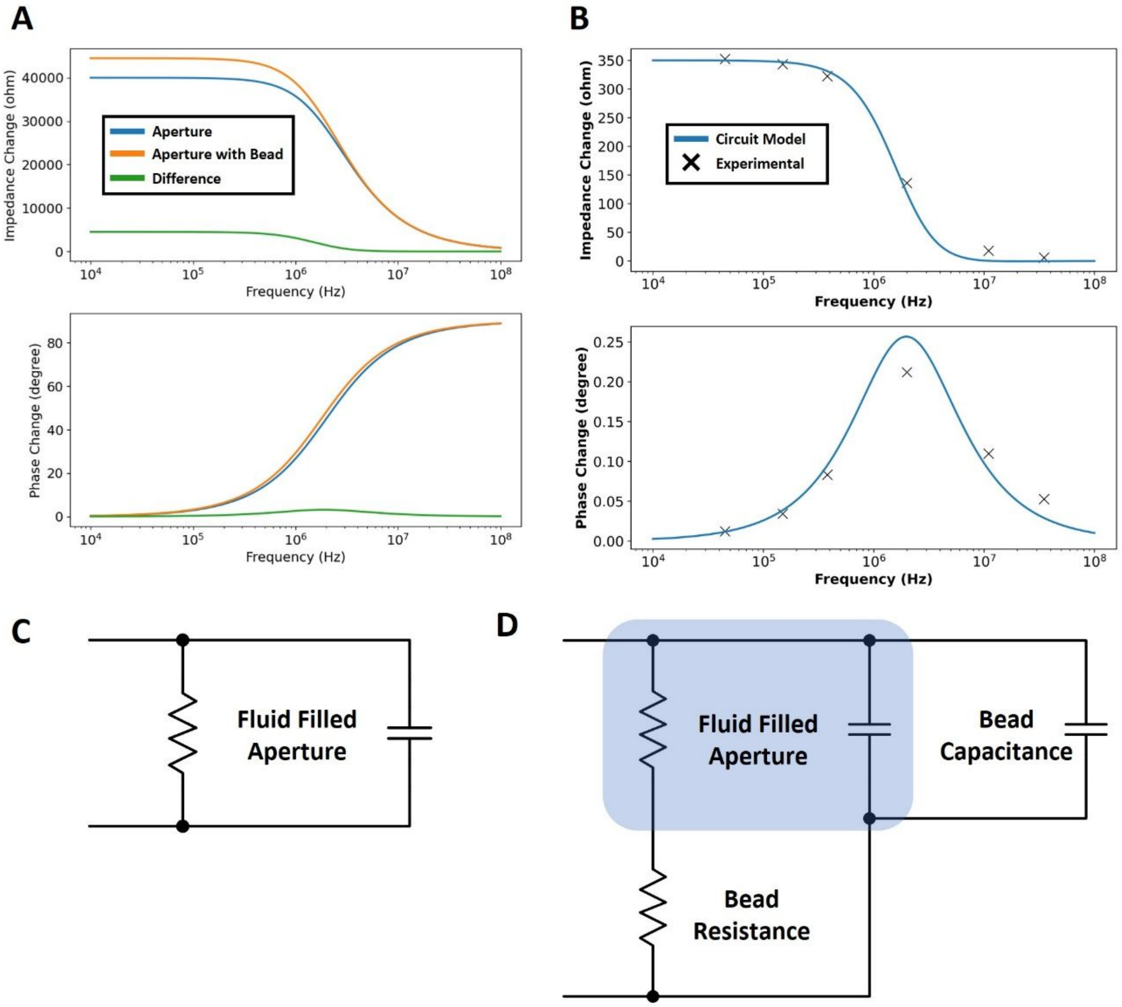

Figure S4: A simple circuit model for non-conducting bead passing through aperture. (a) Bode plots of baseline and response to bead. (b) The circuit model overlayed on experimental data for 8.2 µm bead. (c) Equivalent circuit for aperture. (d) Equivalent circuit for bead passing through aperture.

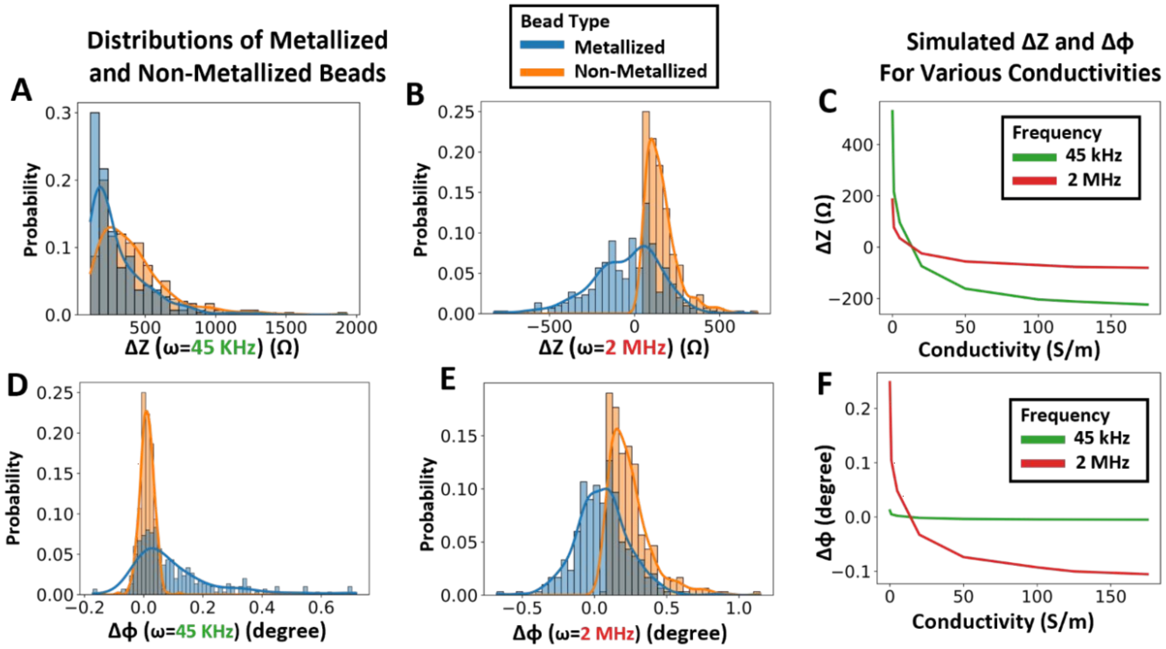

Figure S5: (A,B) Impedance magnitude of non-metallized and metallized beads at 45 kHz and 2 MHz respectively. (C) Simulated impedance magnitude of a bead with metal shell of varying conductivity at 45 kHz and 2 MHz. (D,E,F) Impedance phase plots corresponding to panels a, b, and c respectively.

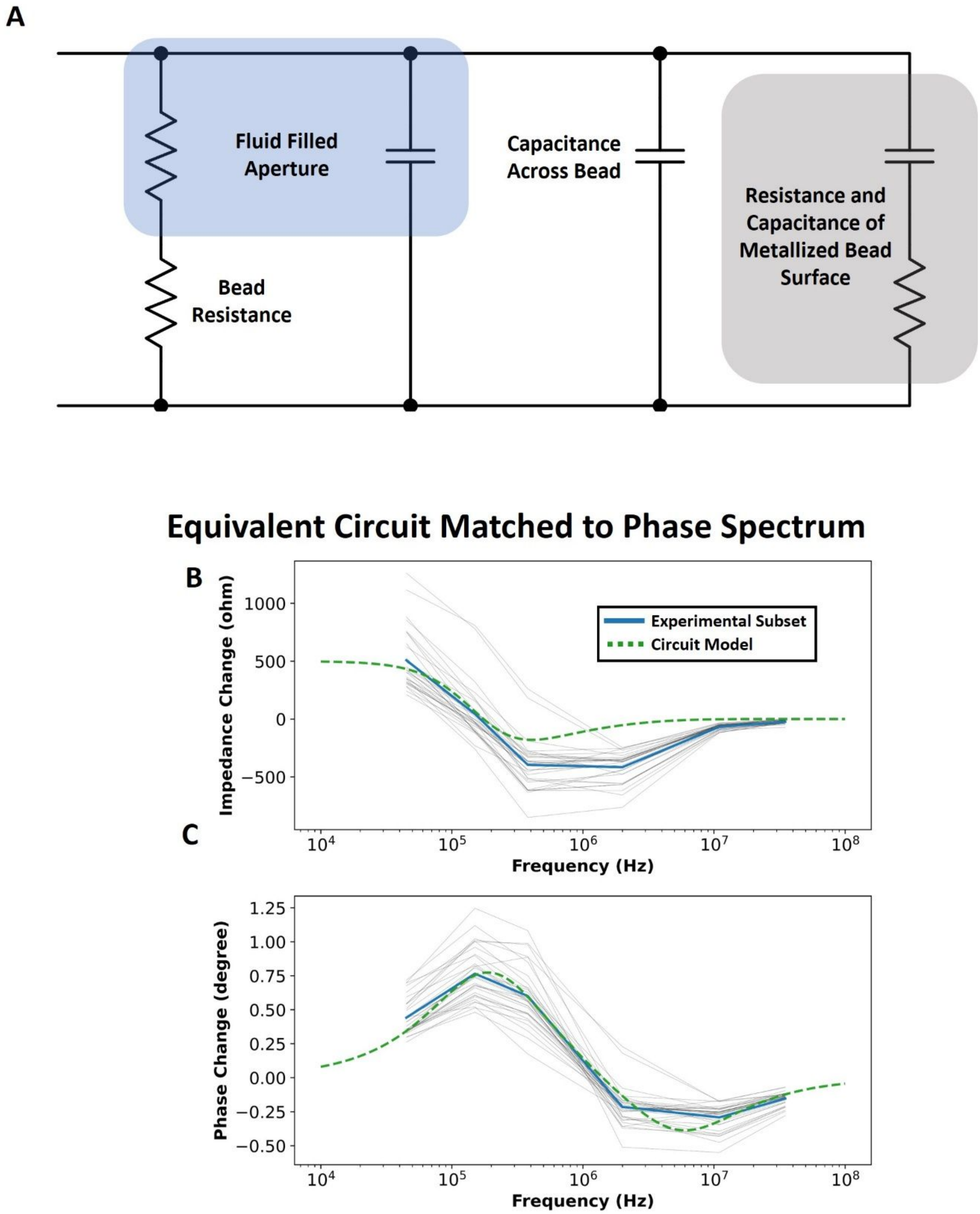

Figure S6: Circuit model for metallized beads meeting the subset criteria in Figure 5 b and e (fuchsia colored) (a) Equivalent circuit. (b-c) Equivalent circuit with components values to match experimental phase peaks.

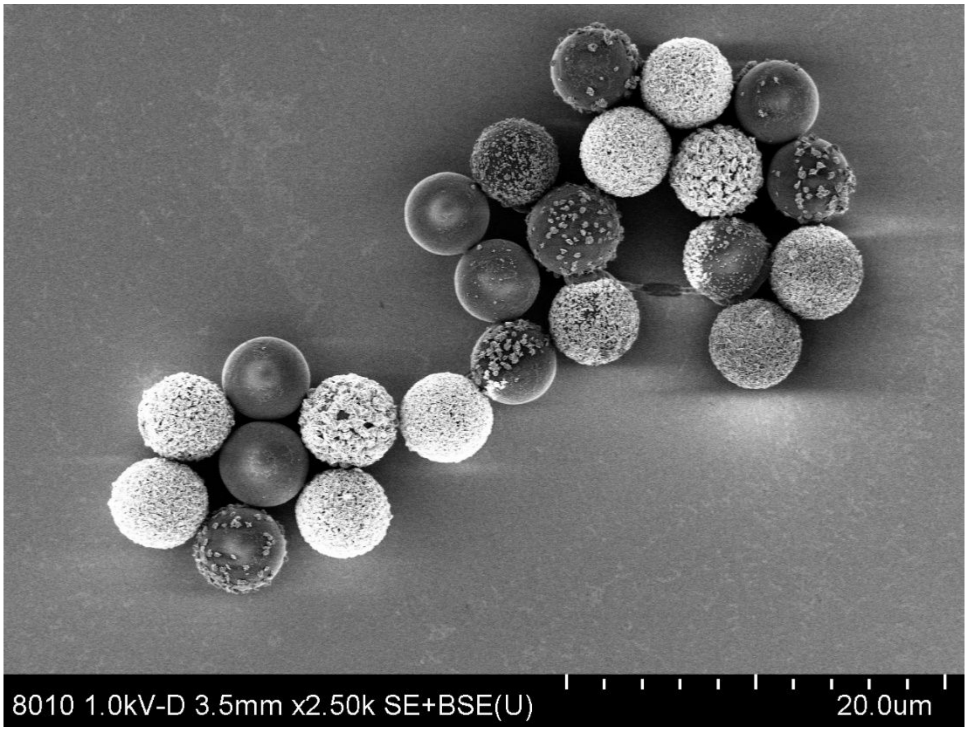

Figure S7: SEM image of several beads showing their non-regular amounts of metallization on 5.7 um beads. Note that non-magnetic beads with little metallization have much smoother surfaces than those of bare magnetic beads such as in Figure 1B.
